# Supplementary figures and images for: Transient Enlargement in Meningiomas Treated with Stereotactic Radiotherapy
Source: Cancers (Basel). 2022 Mar 17;14(6):1547. doi: 10.3390/cancers14061547 (PMC8946188; doi:10.3390/cancers14061547)

**Figure S1:** Measured tumor volumes over time for all patients

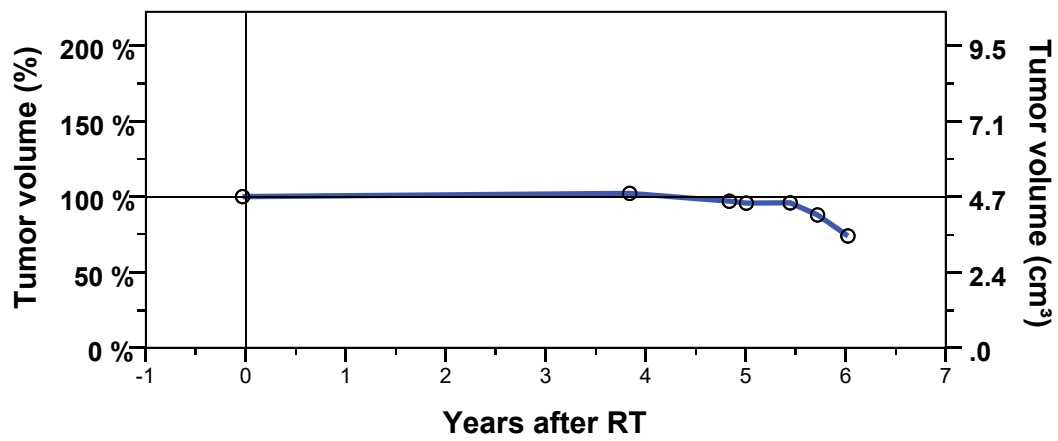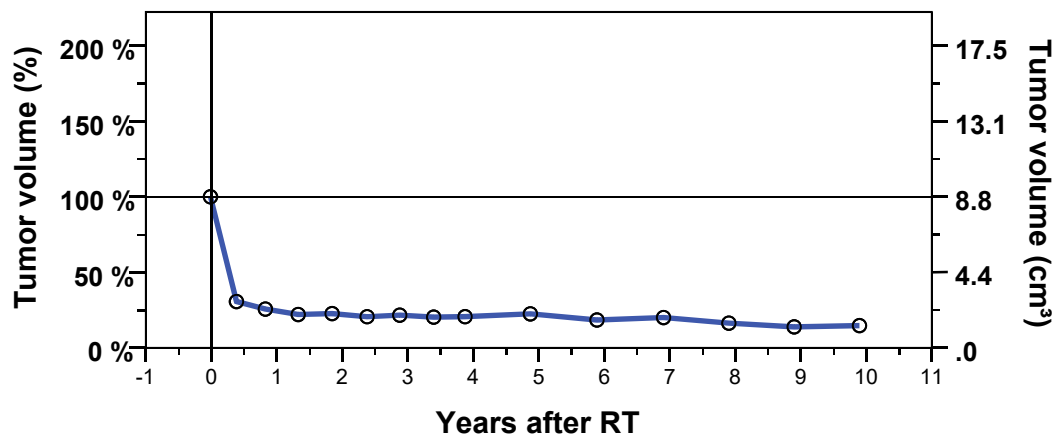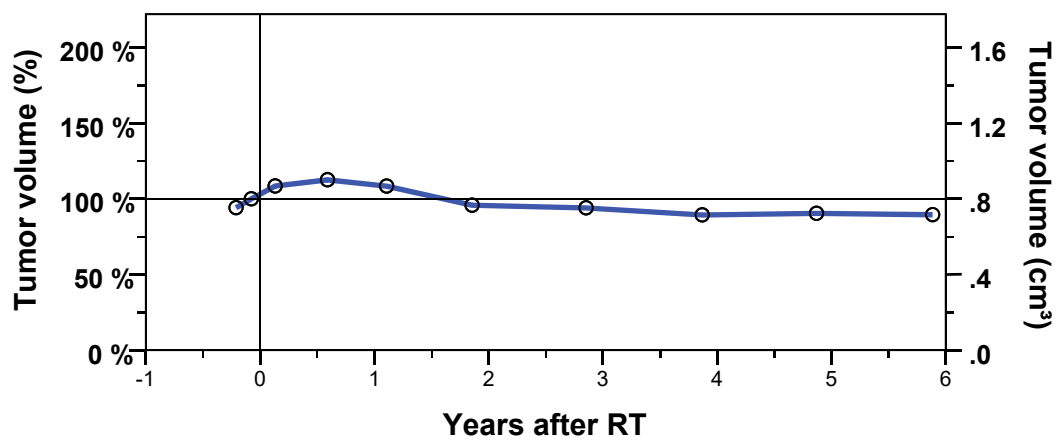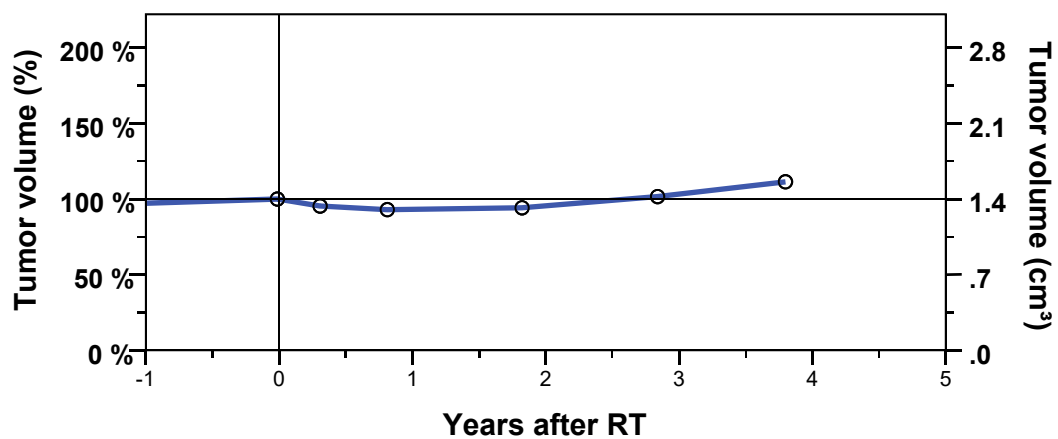

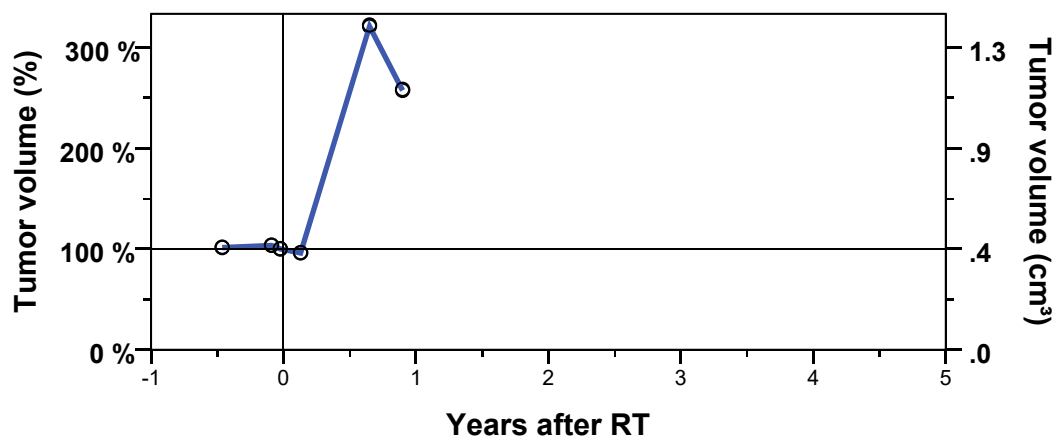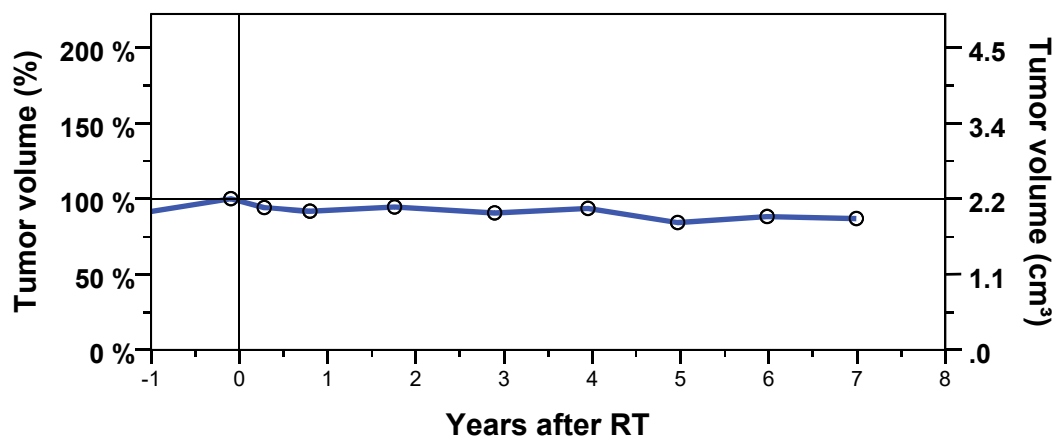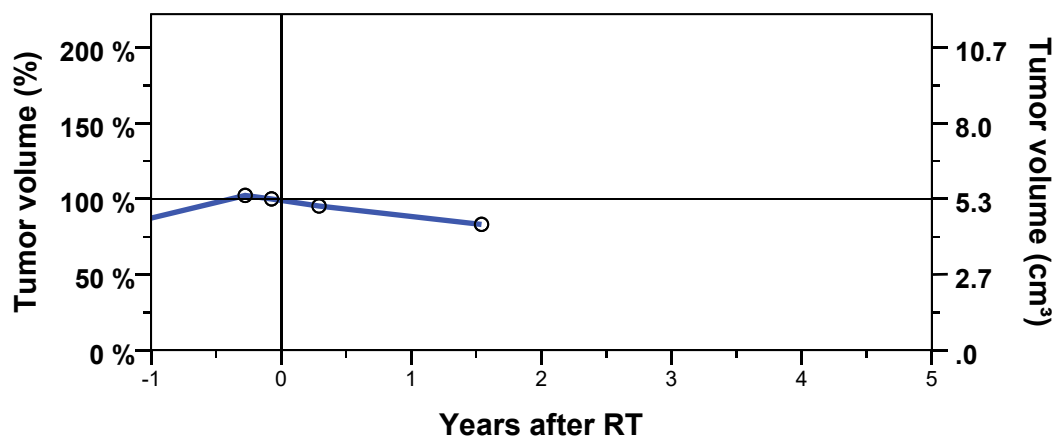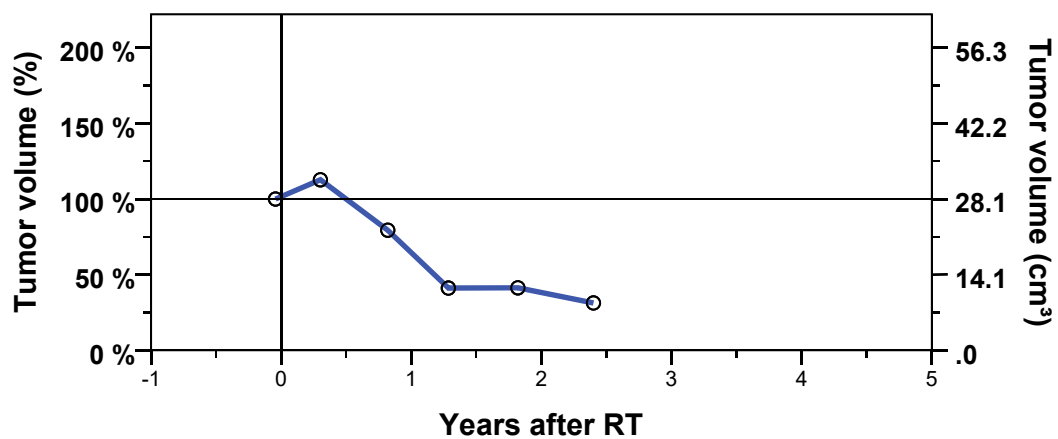

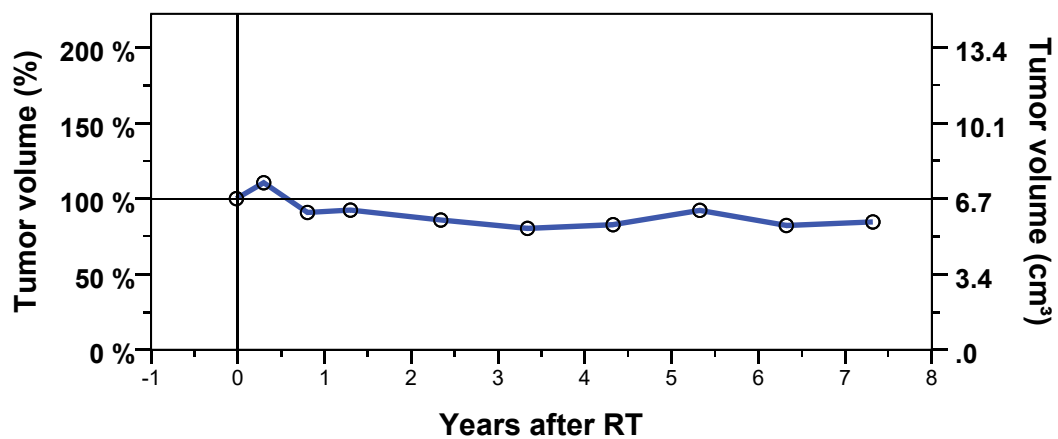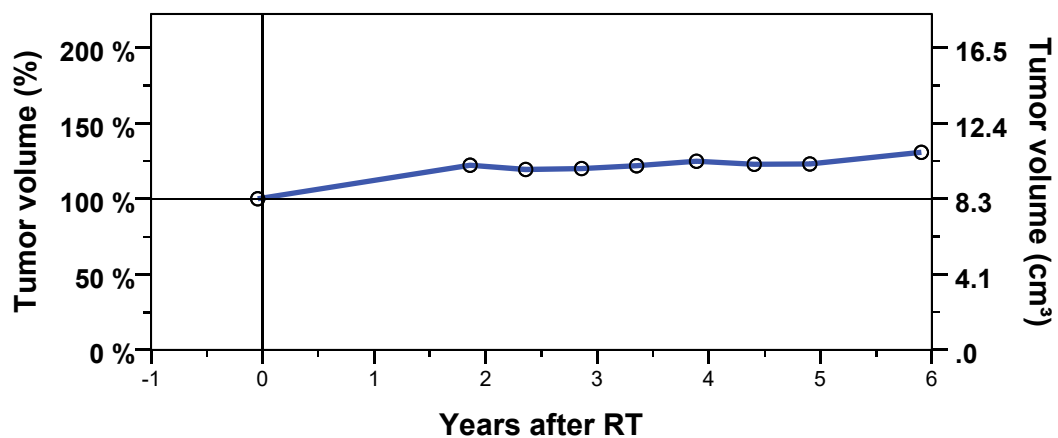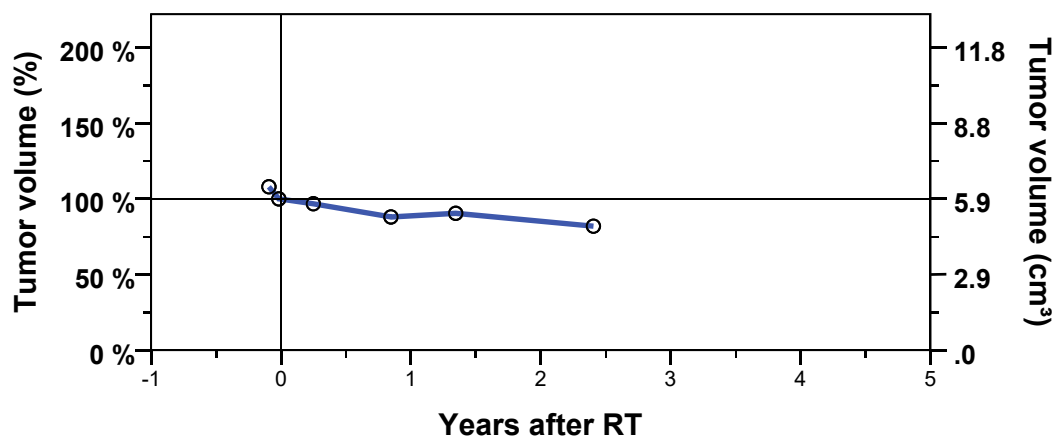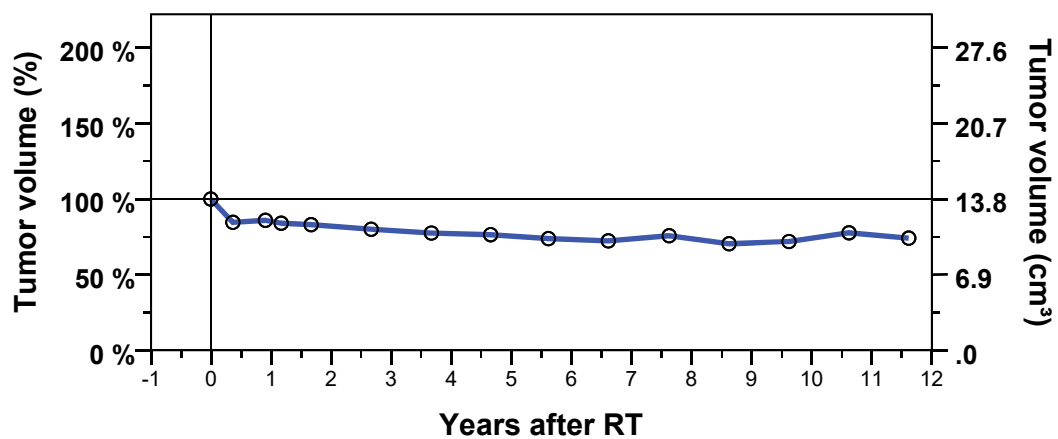

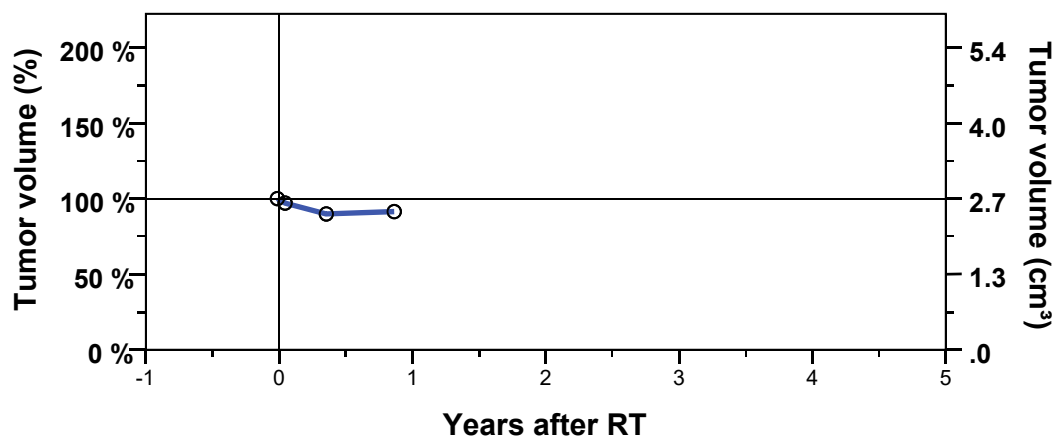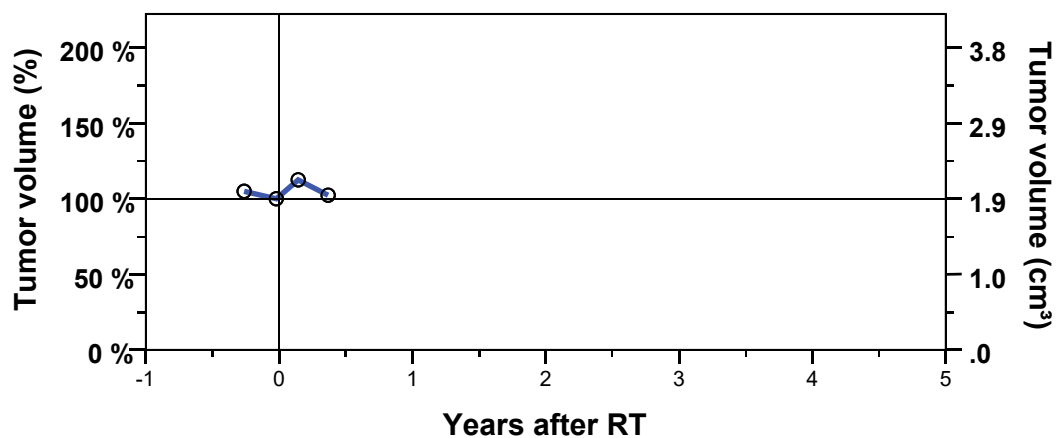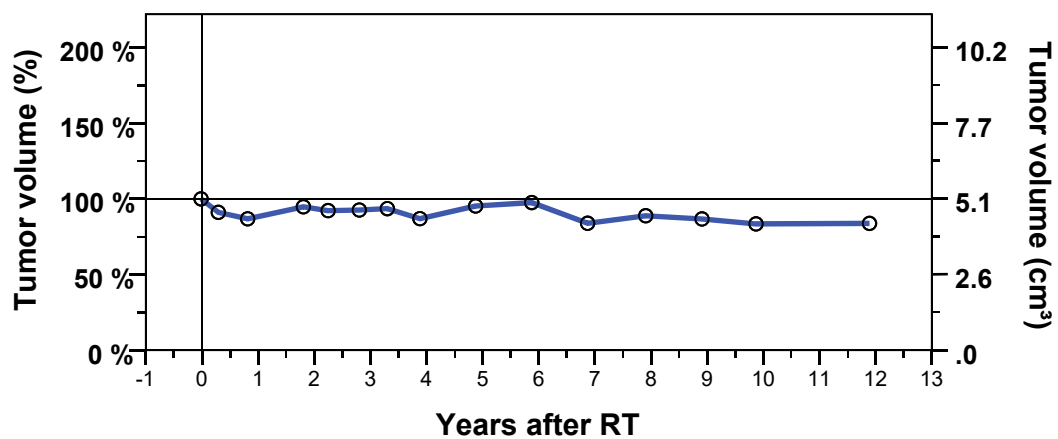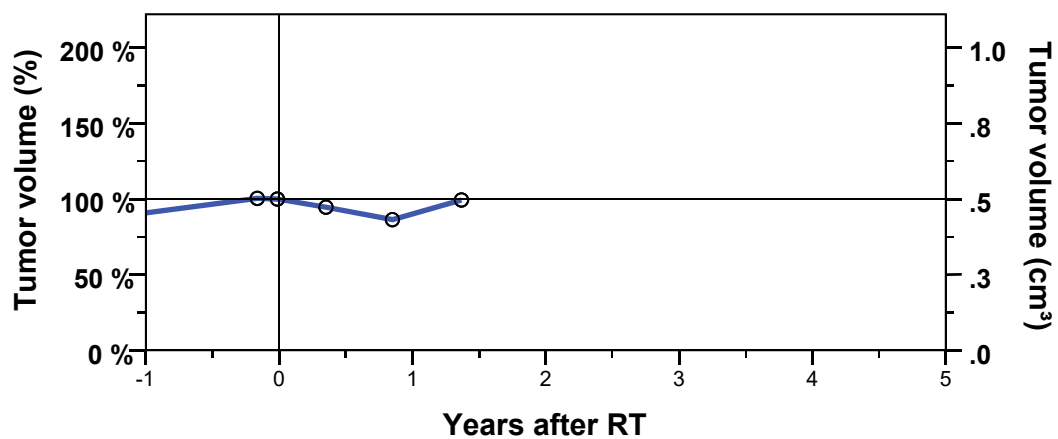

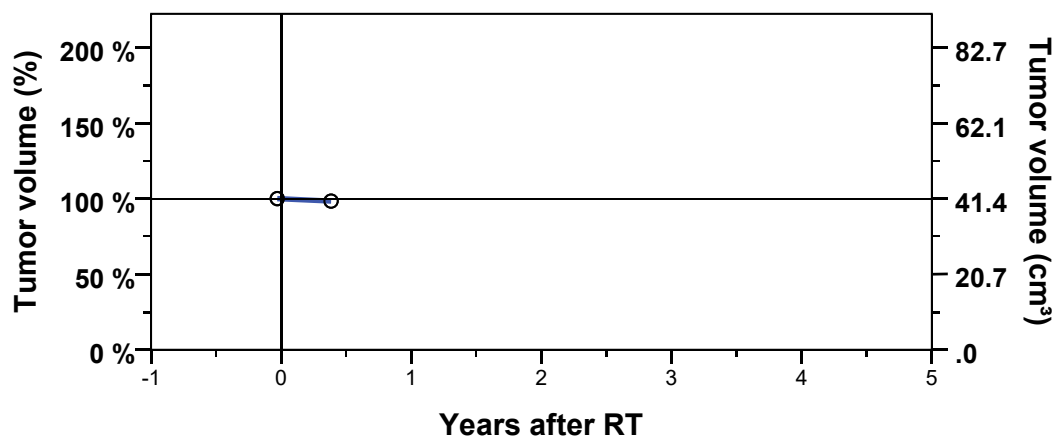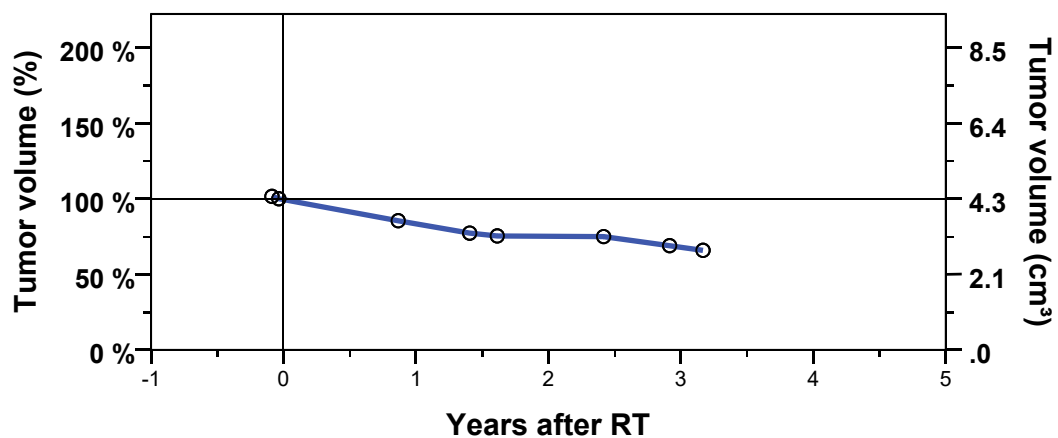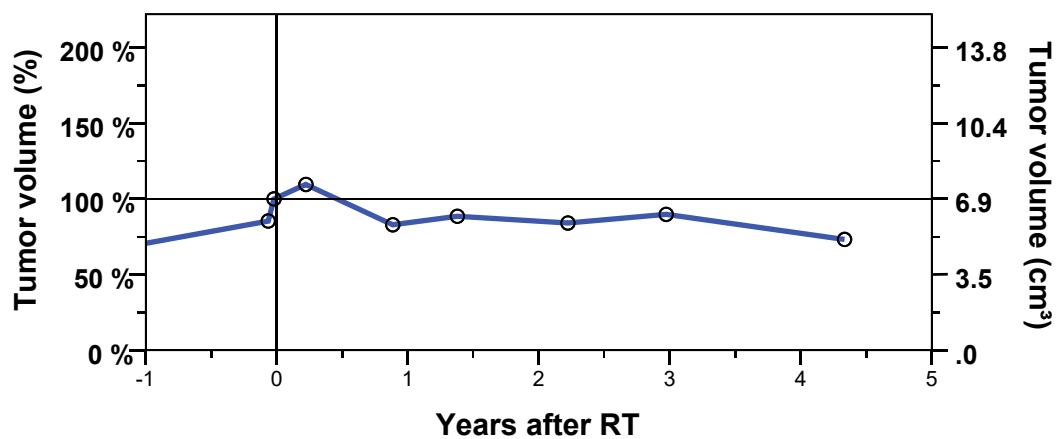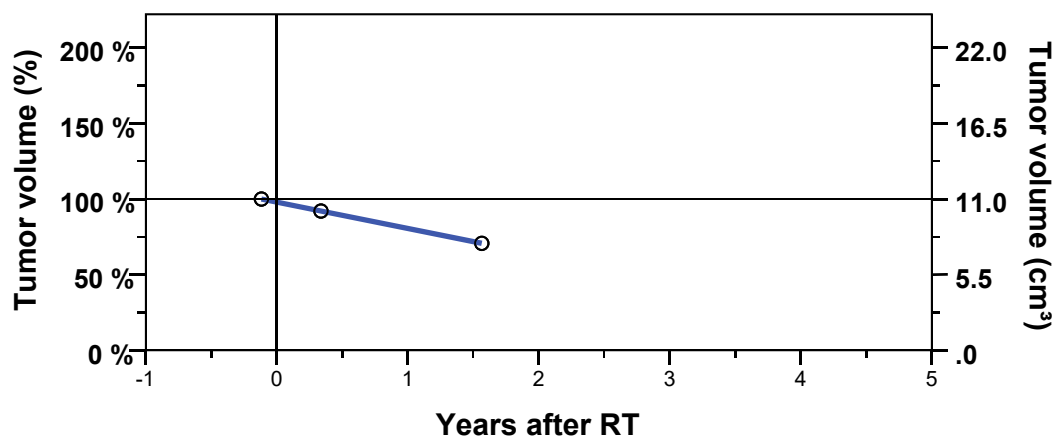

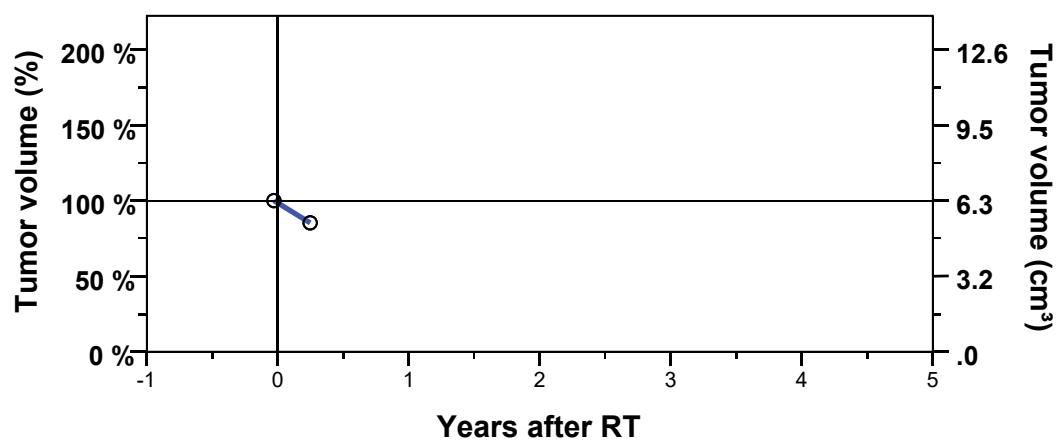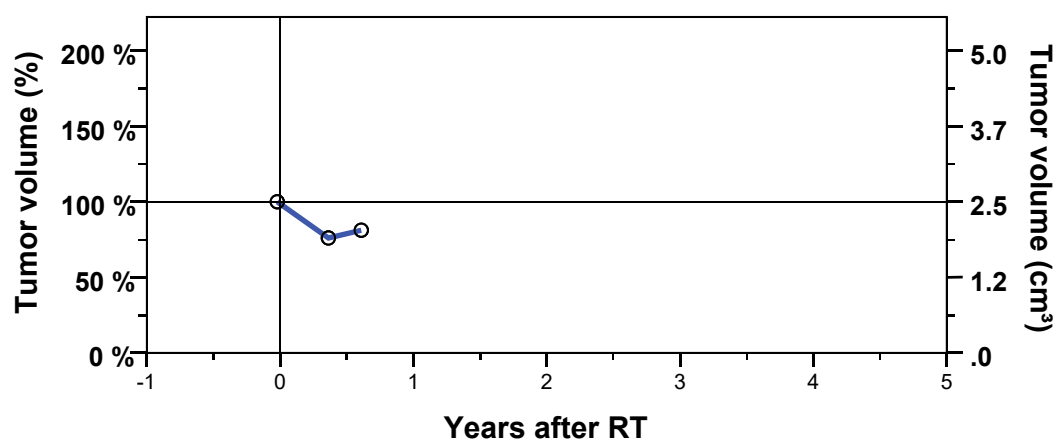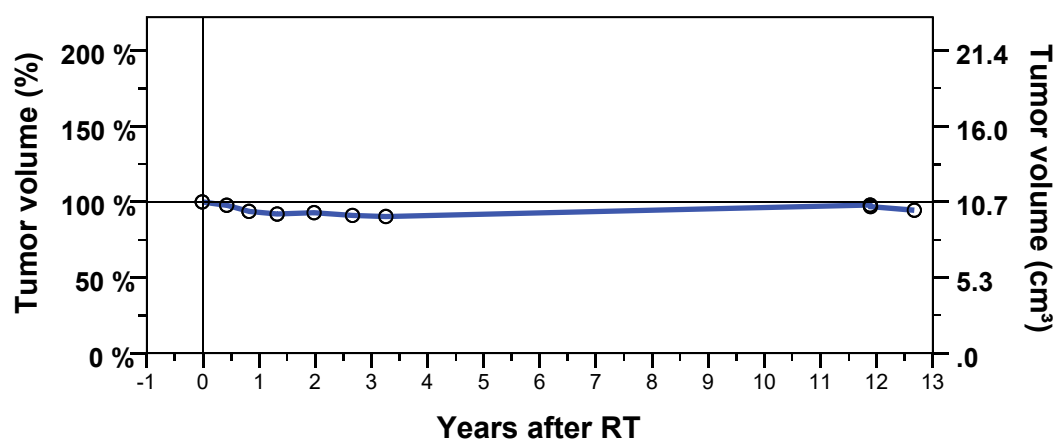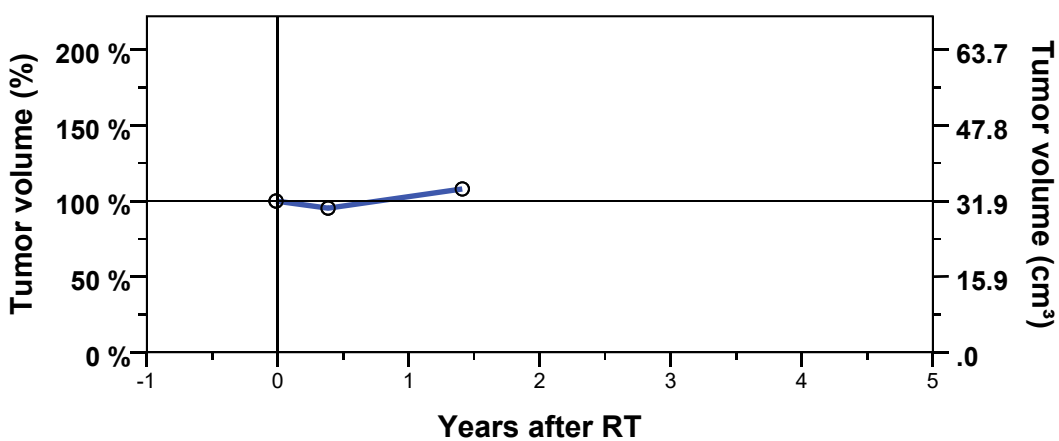

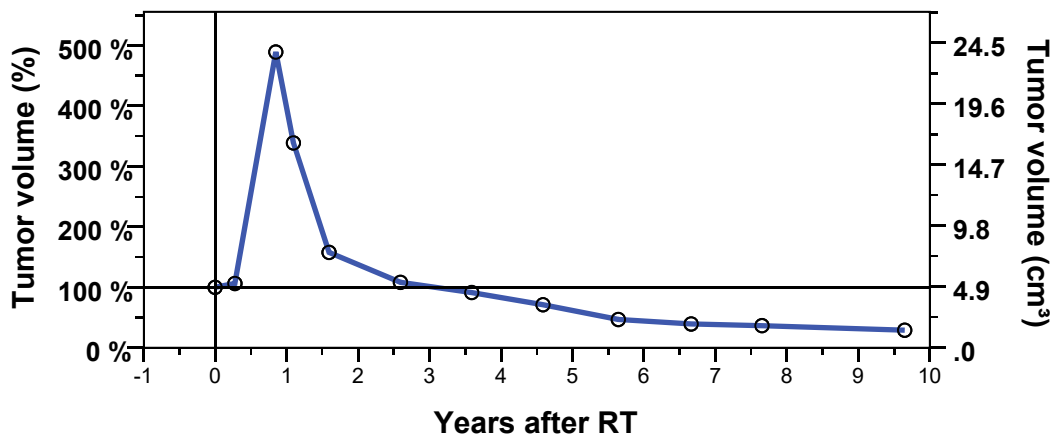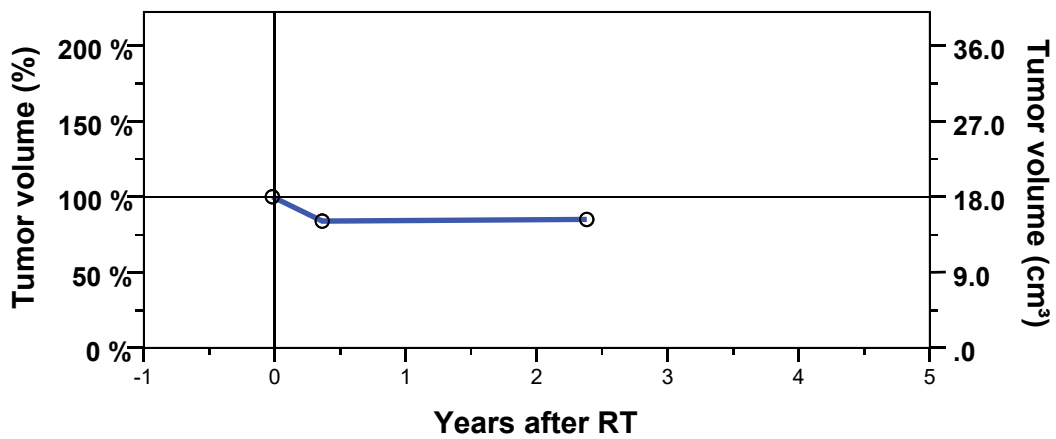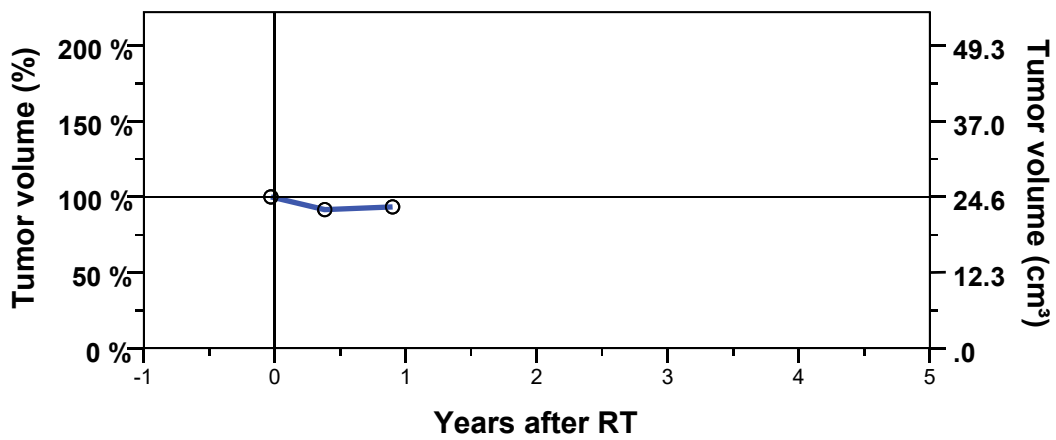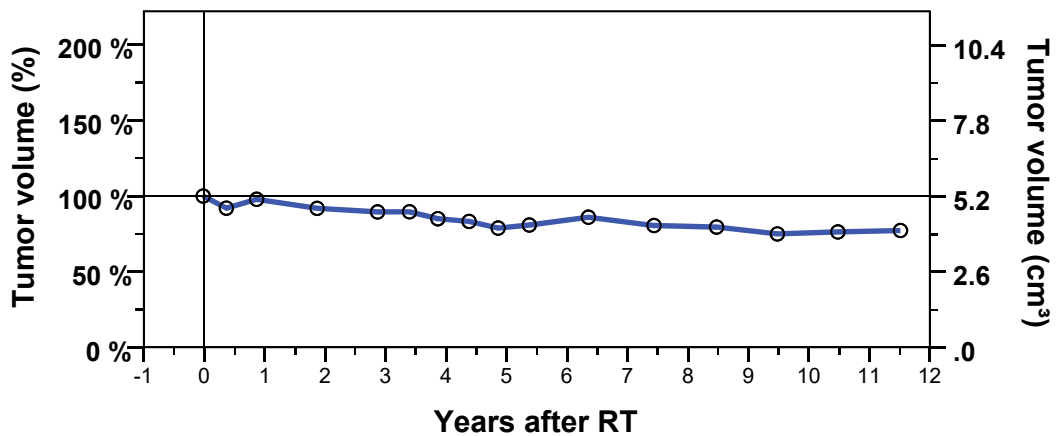

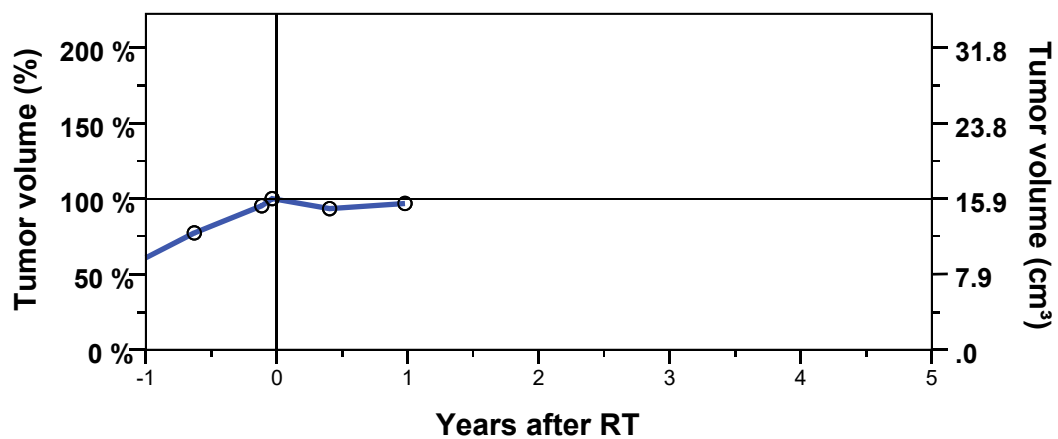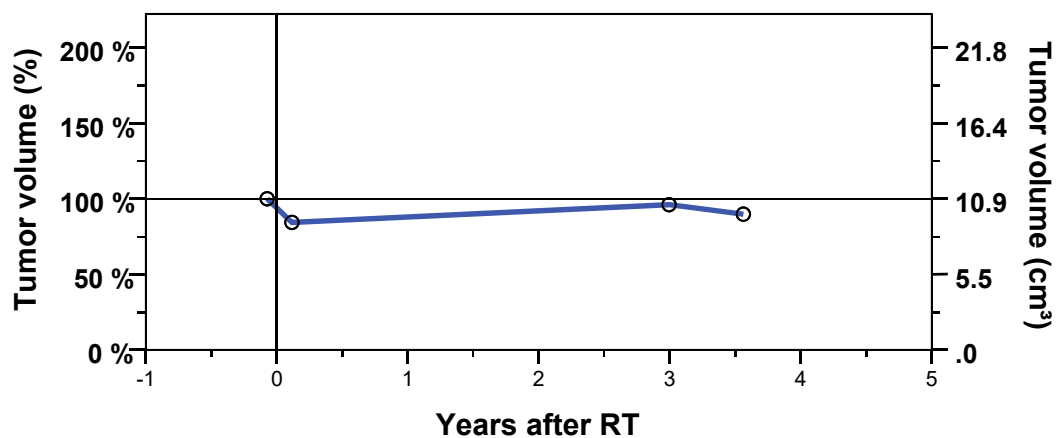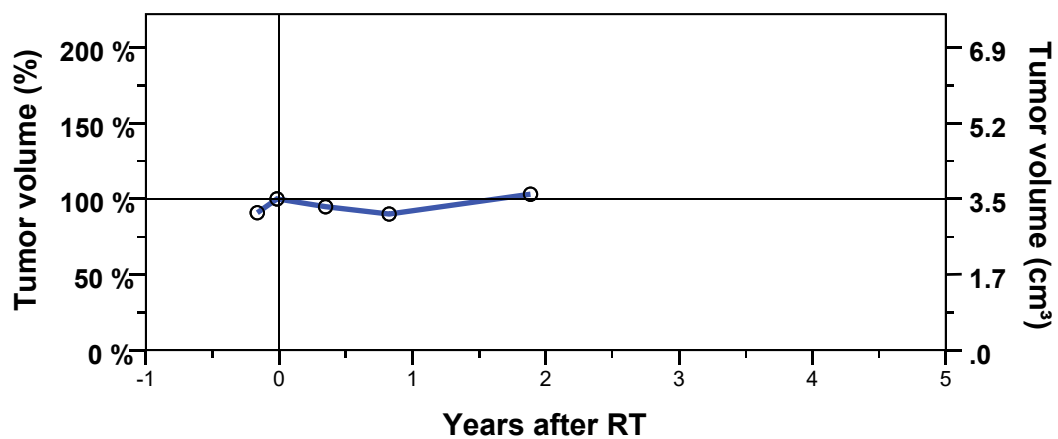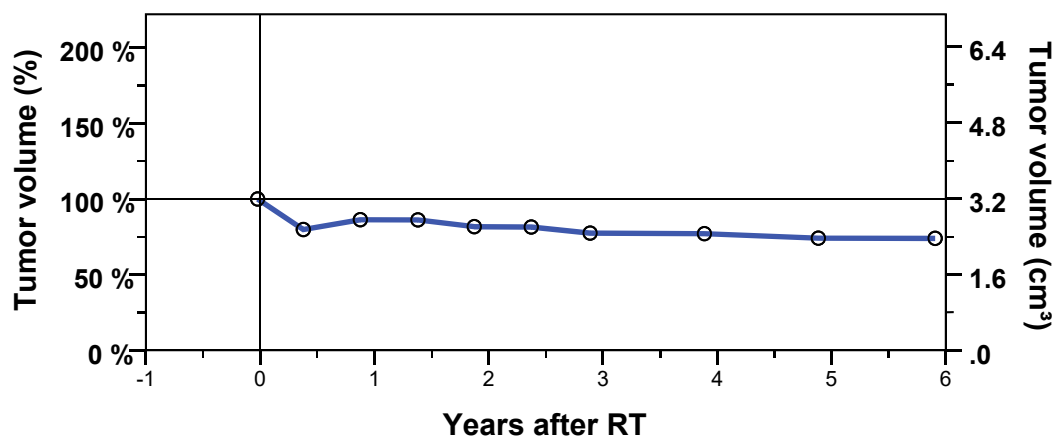

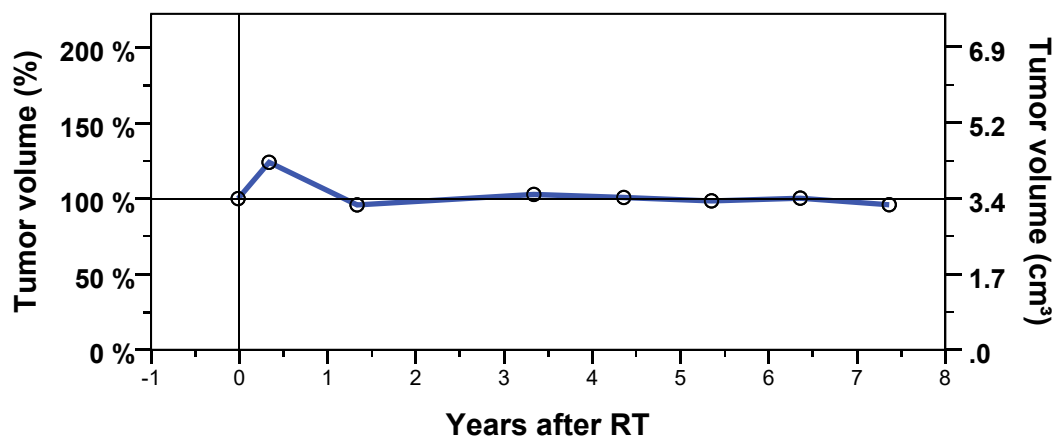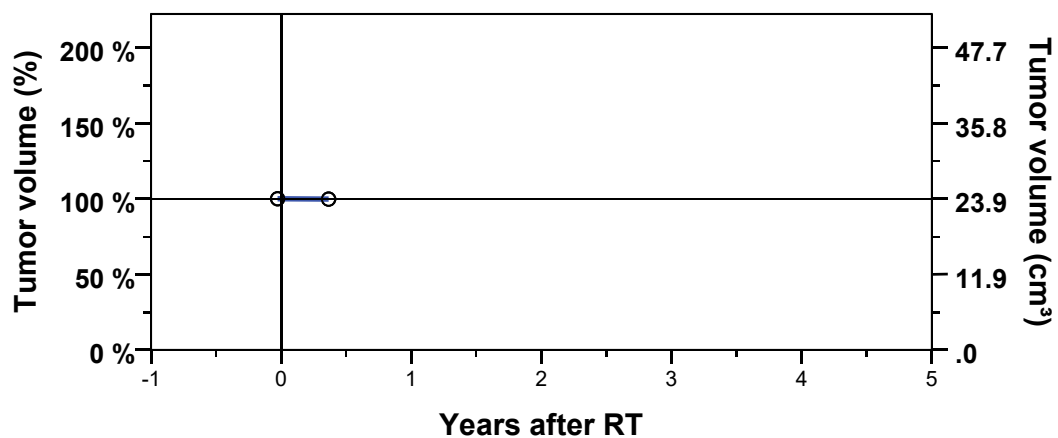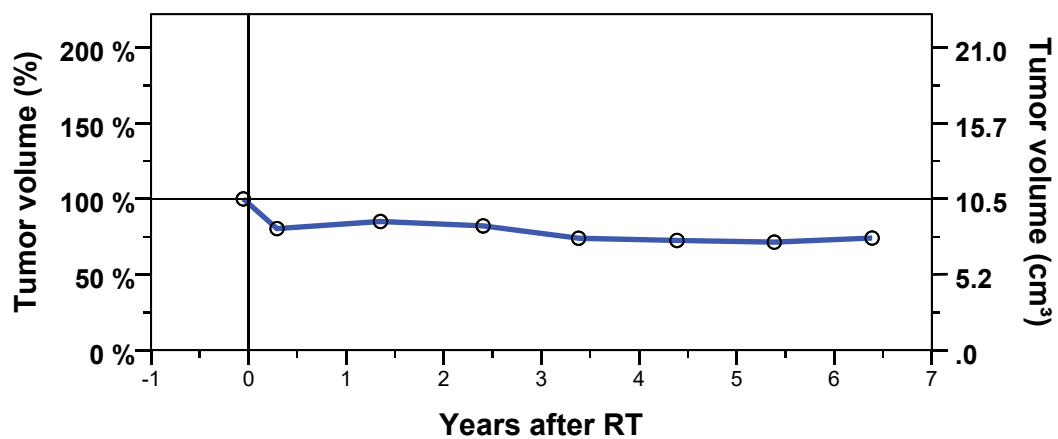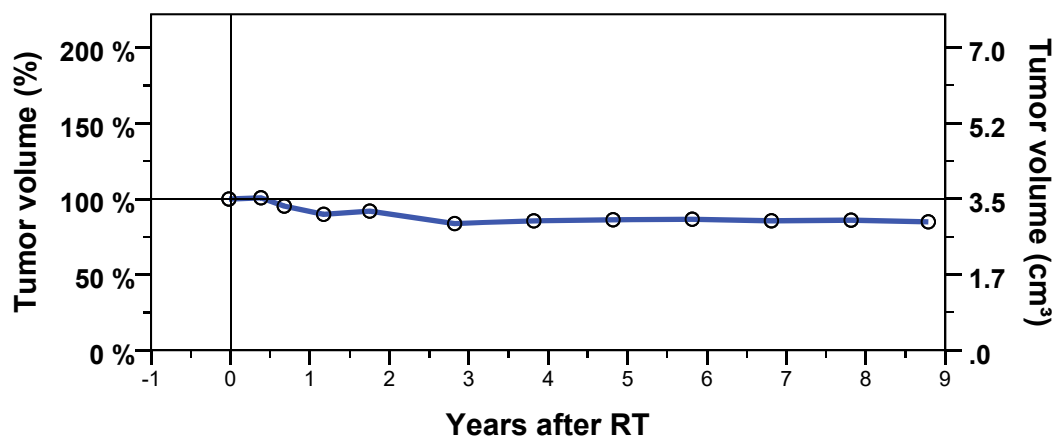

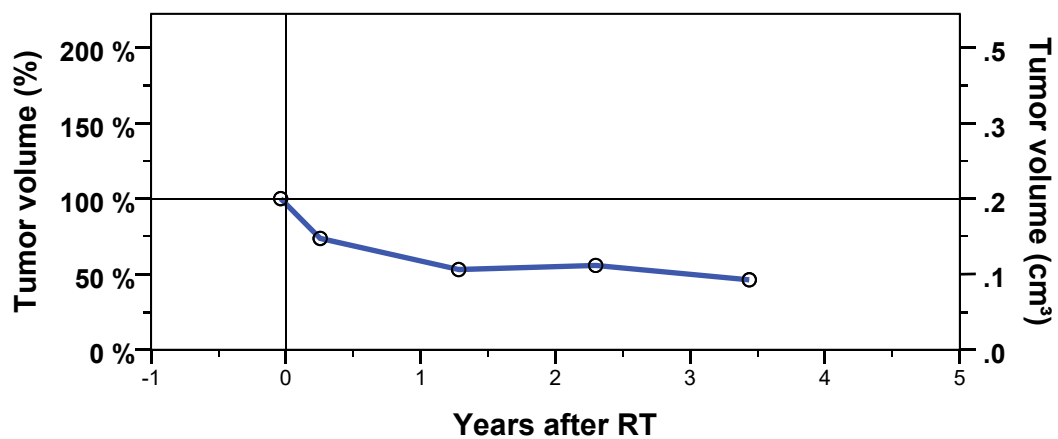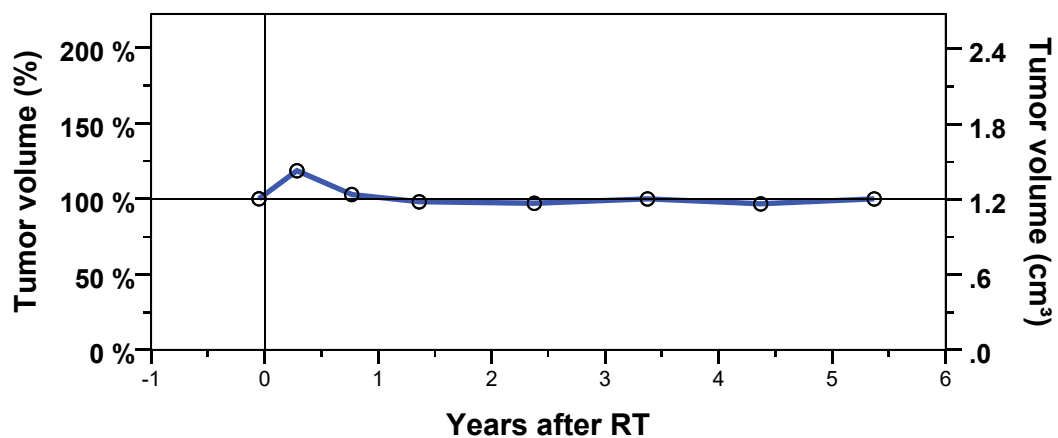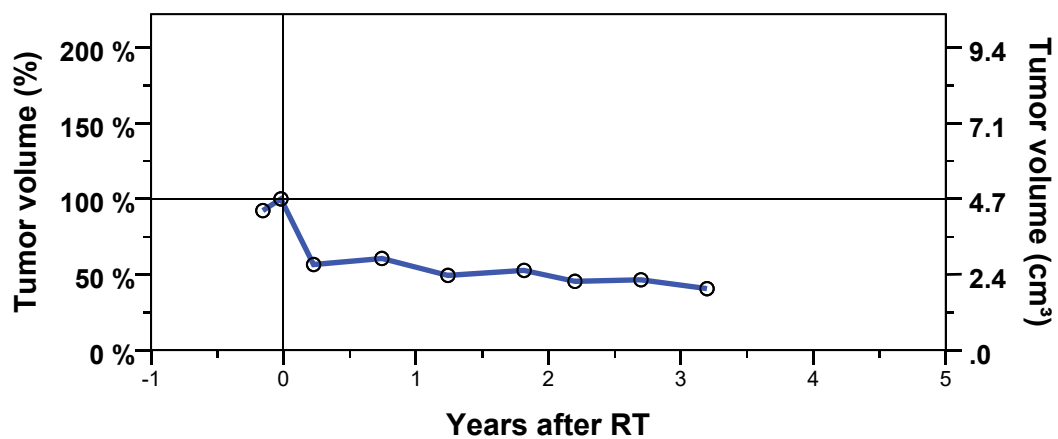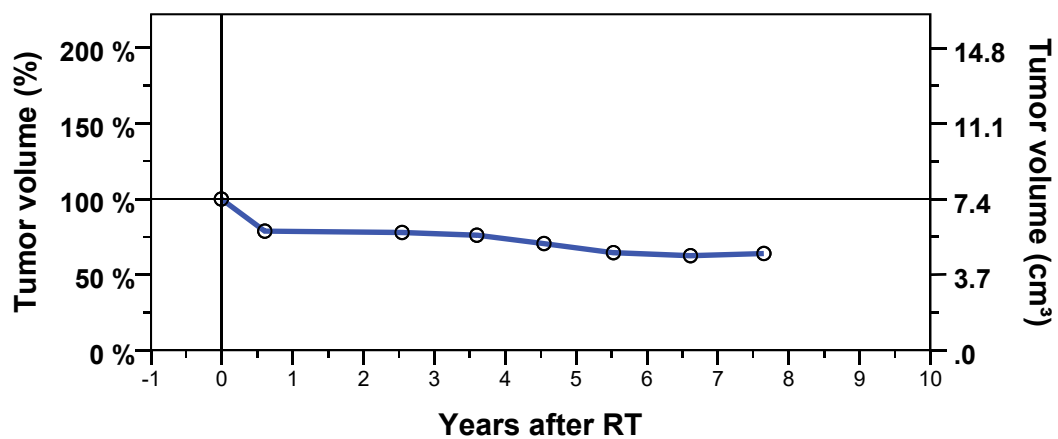

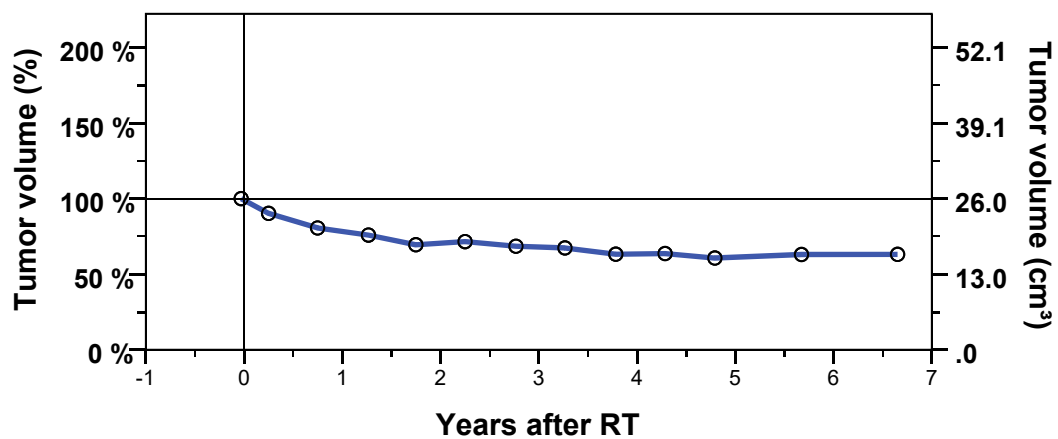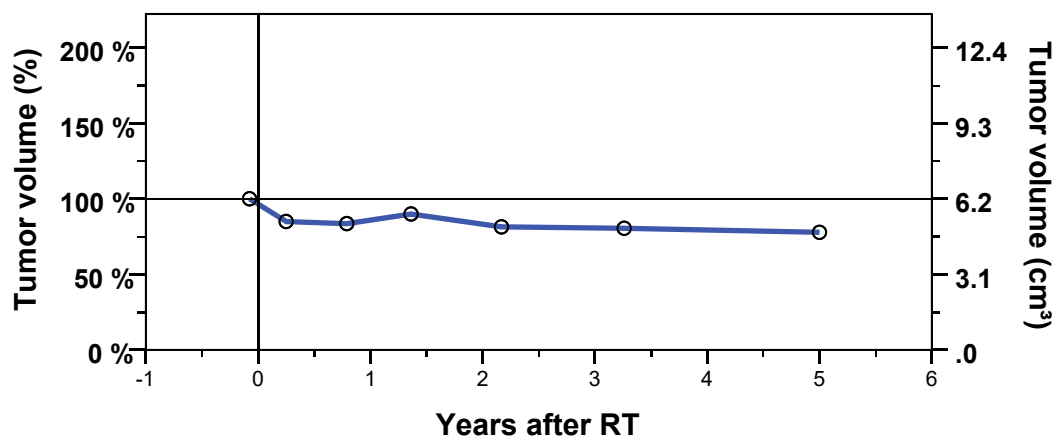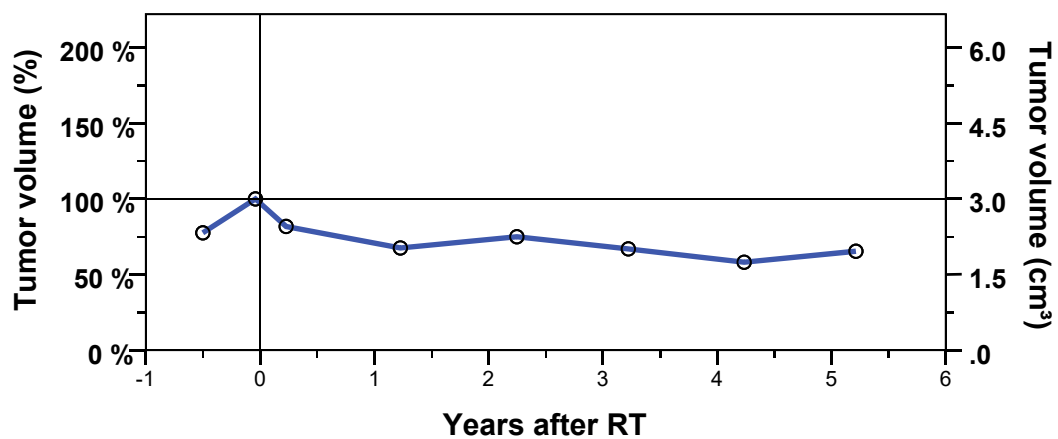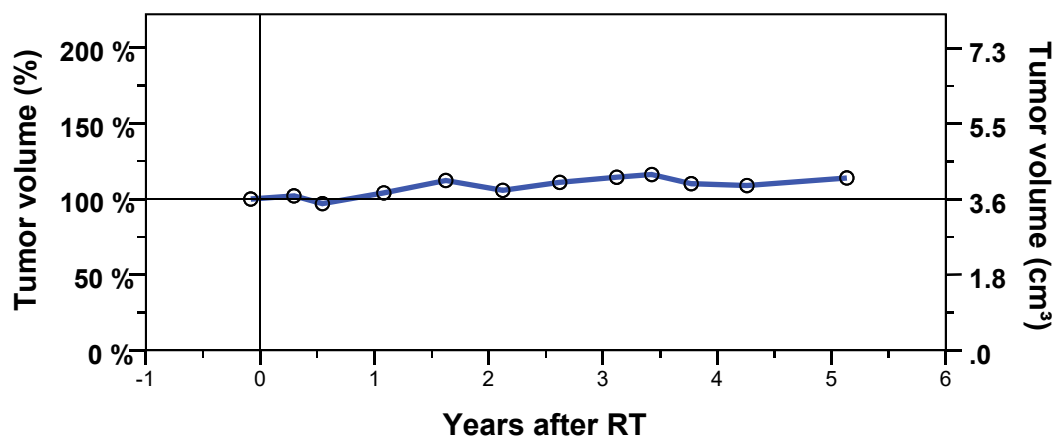

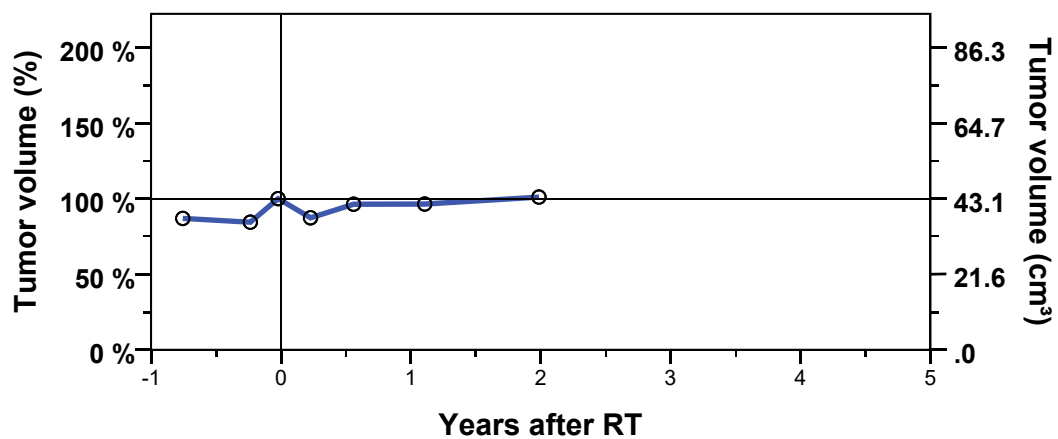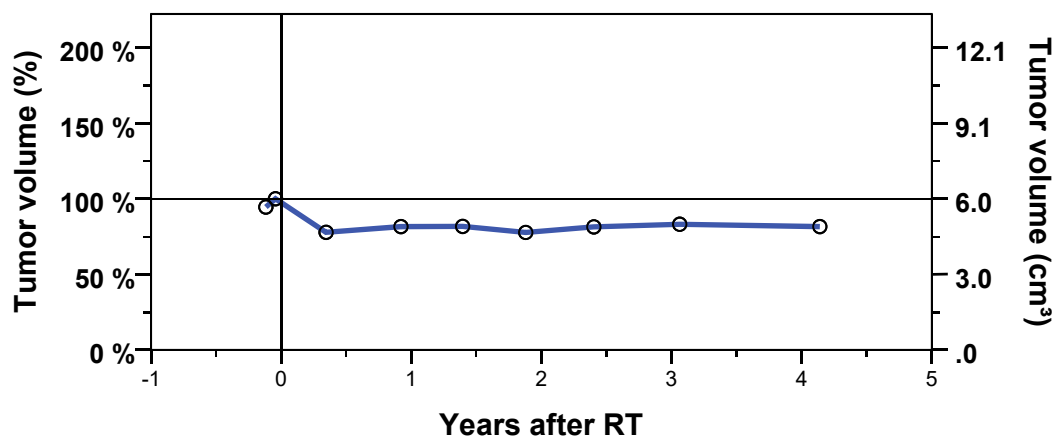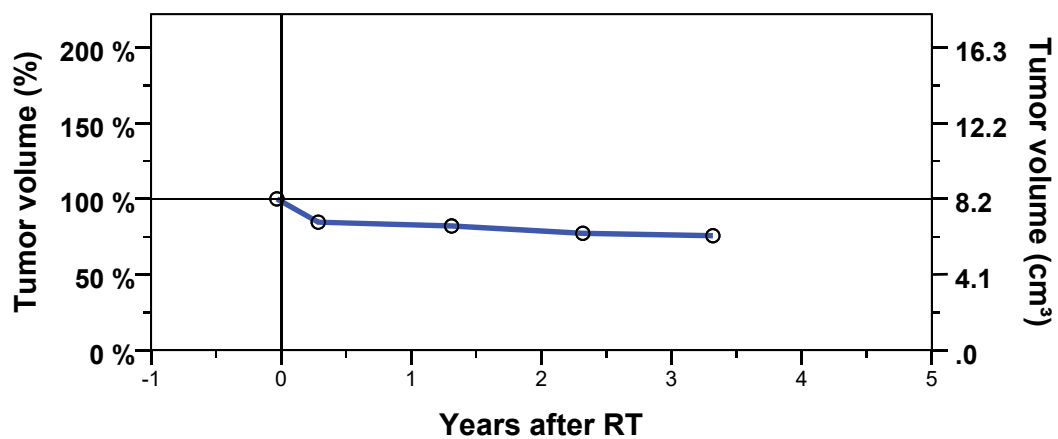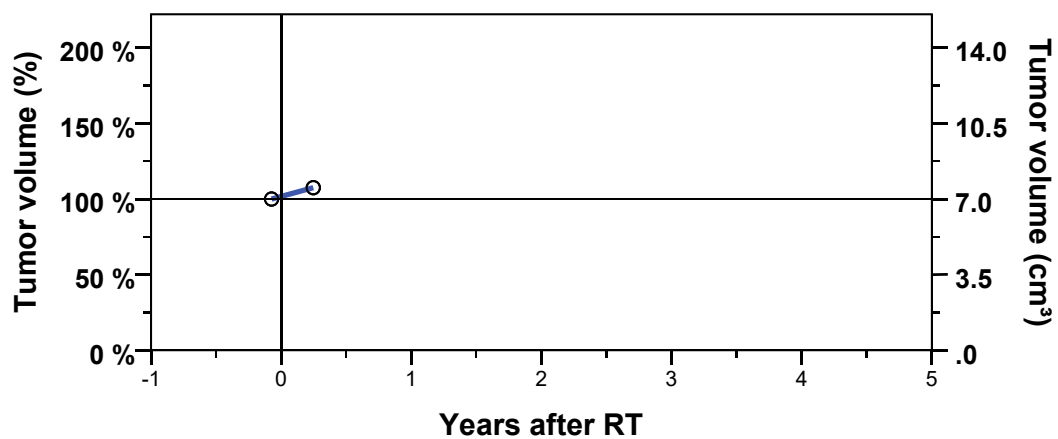

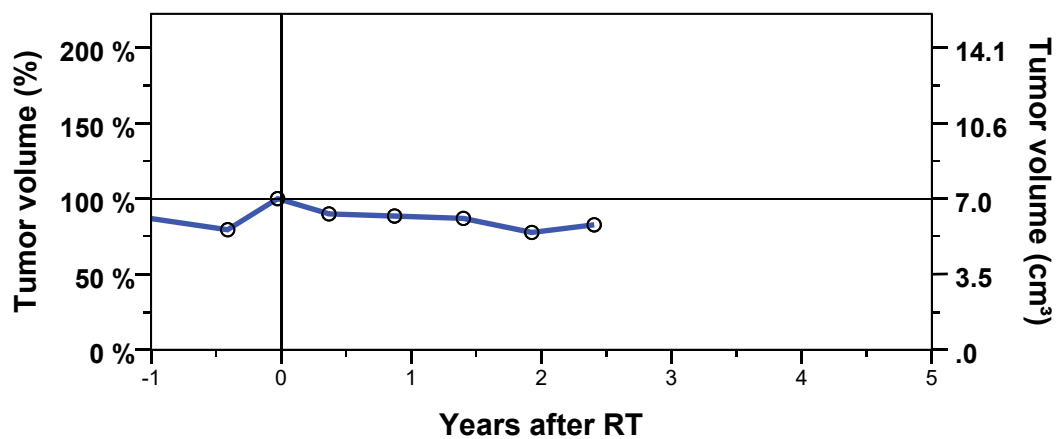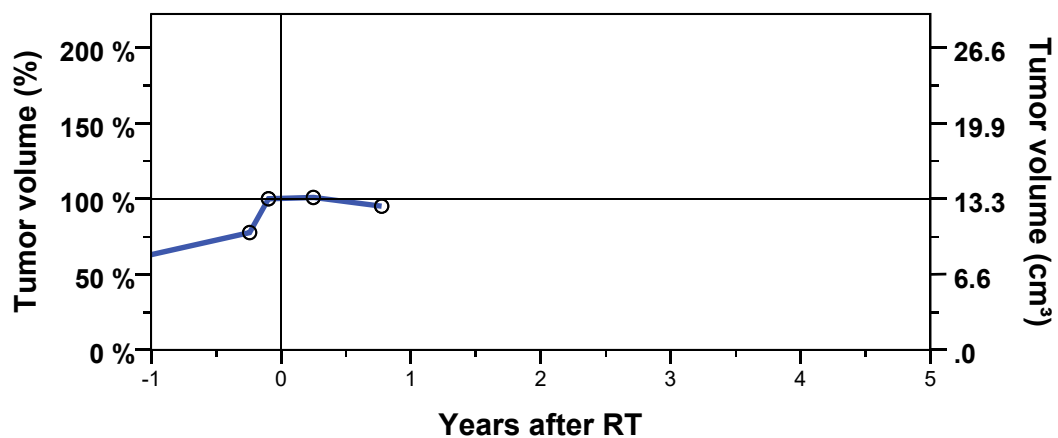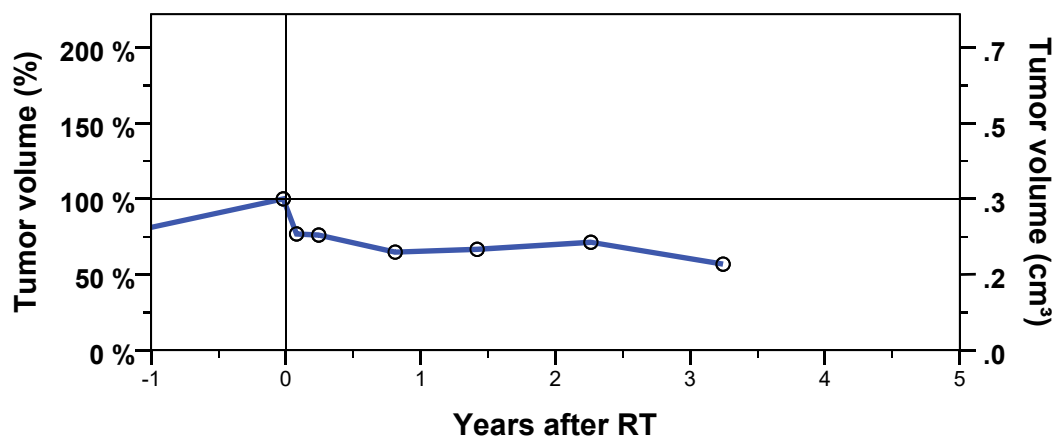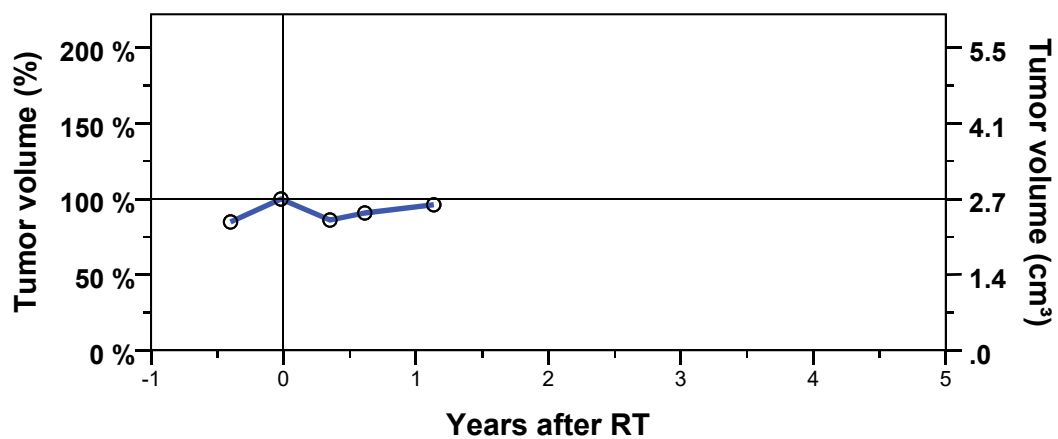

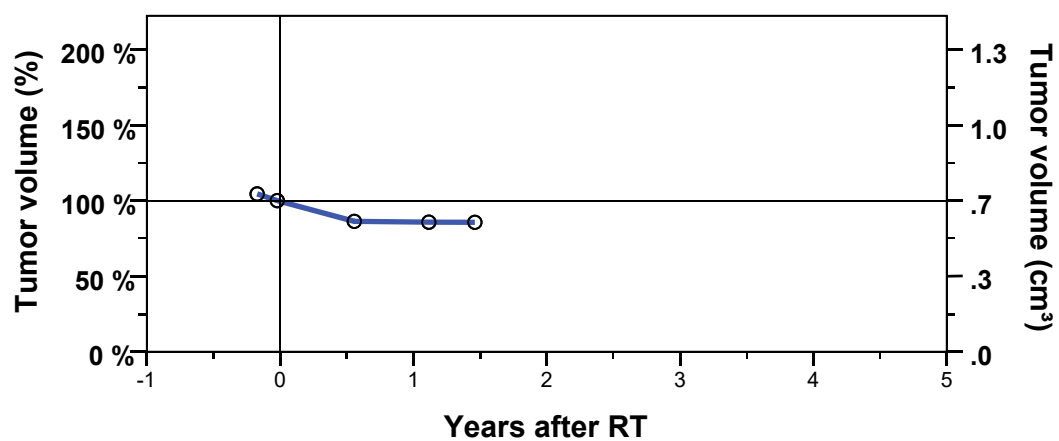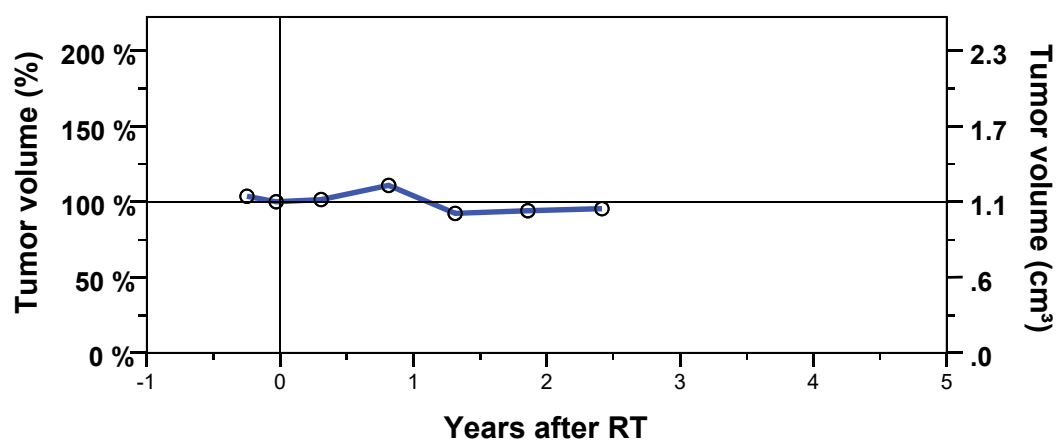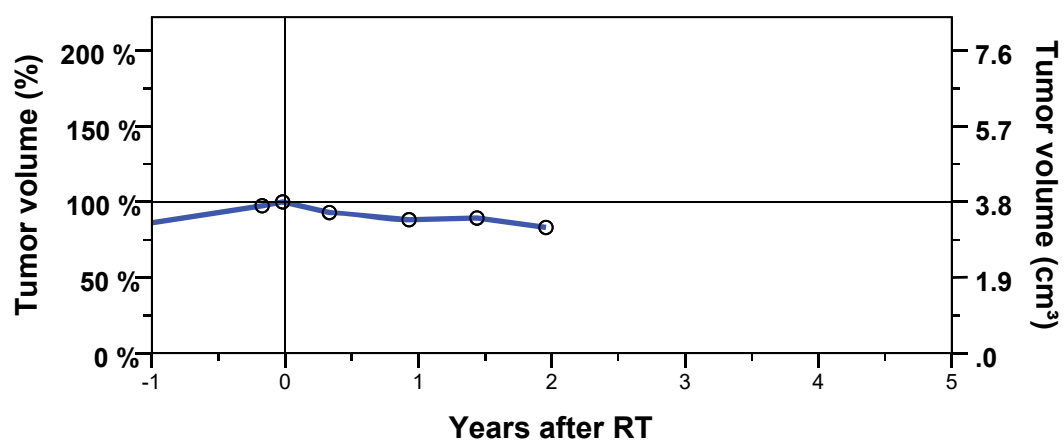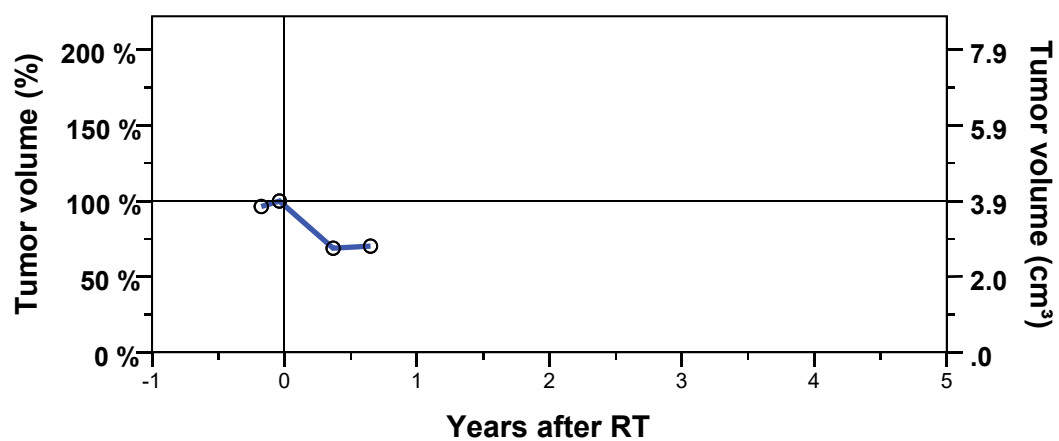

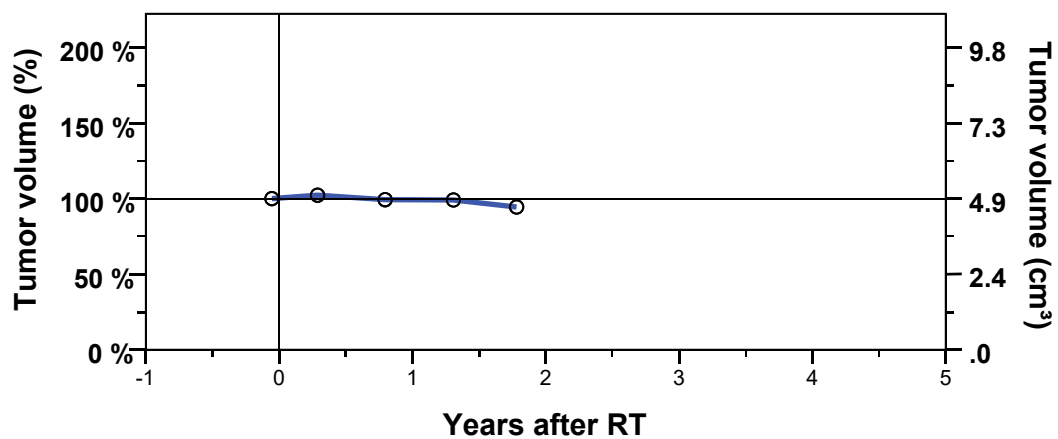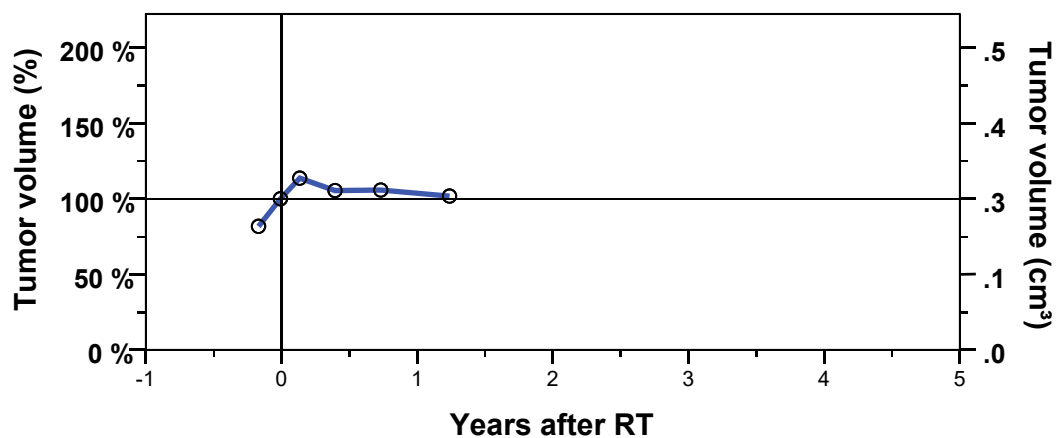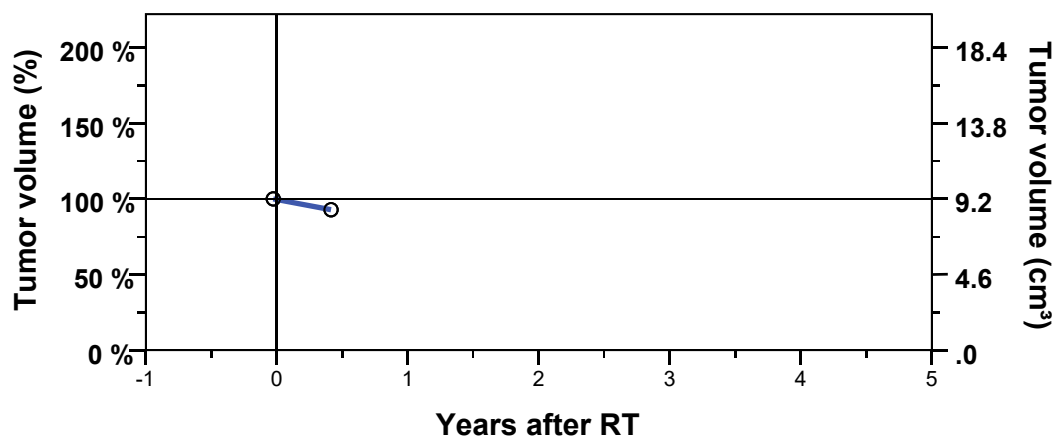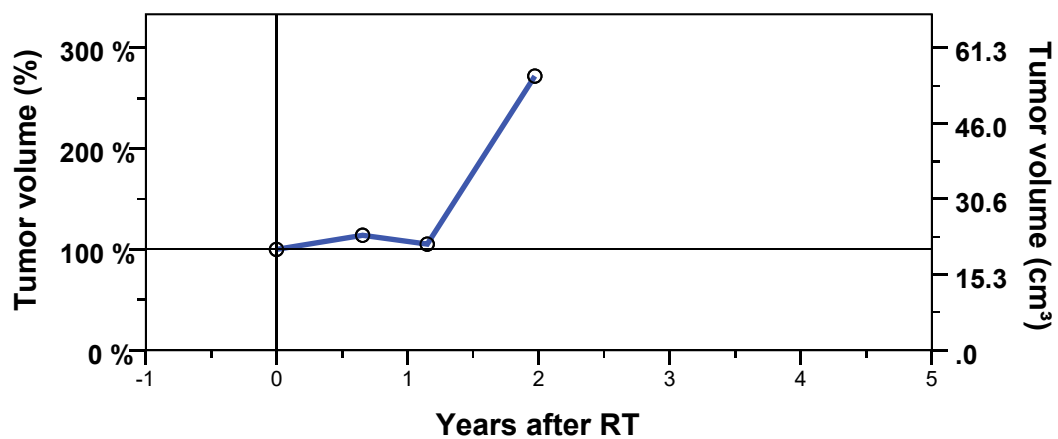

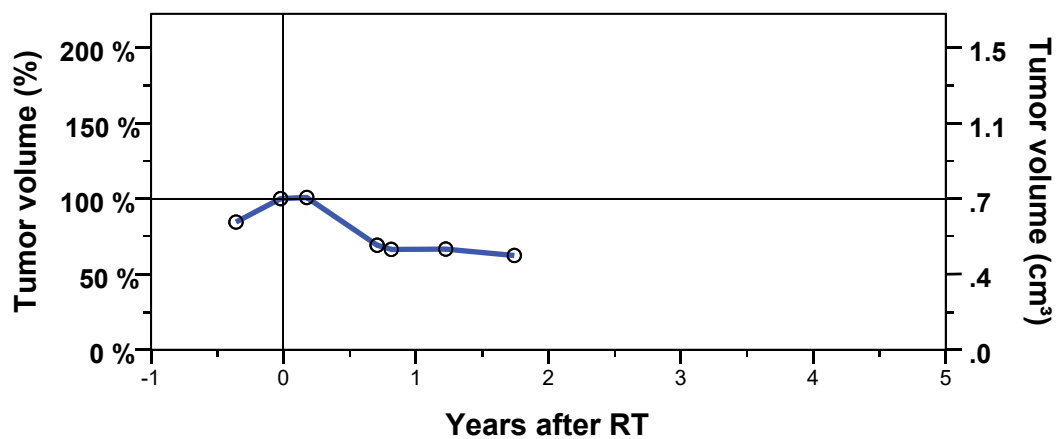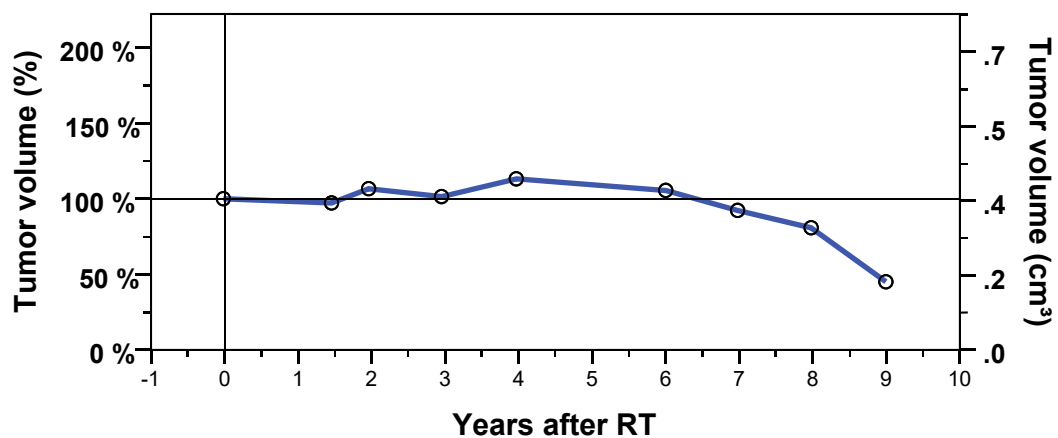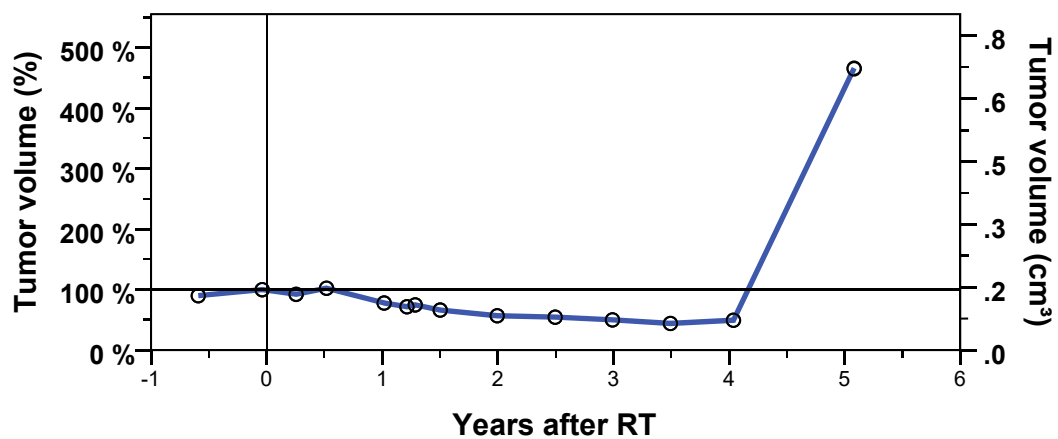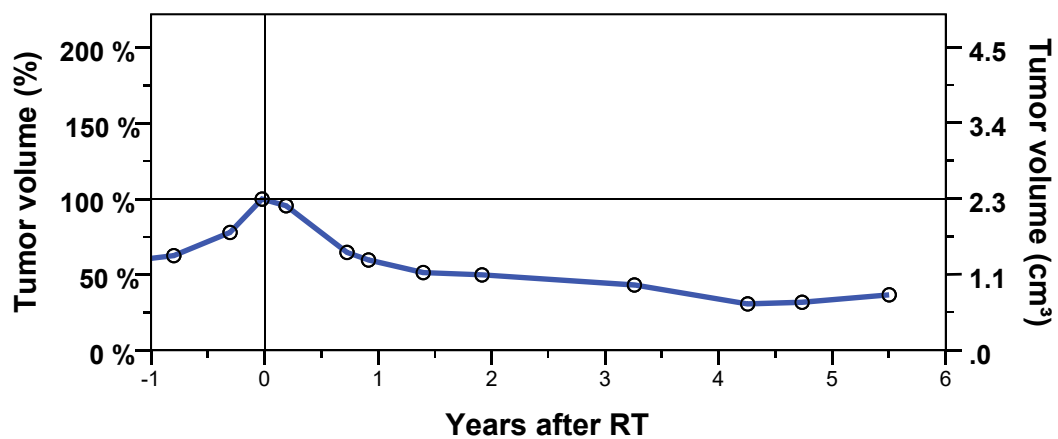

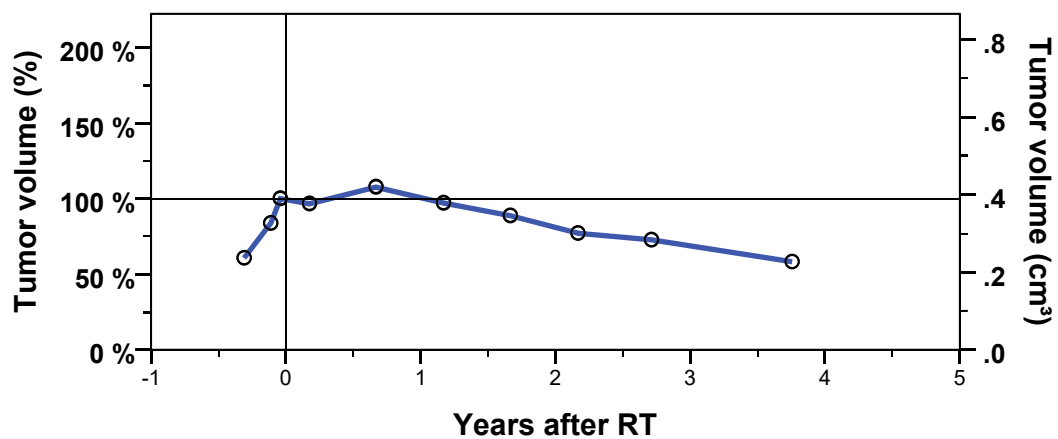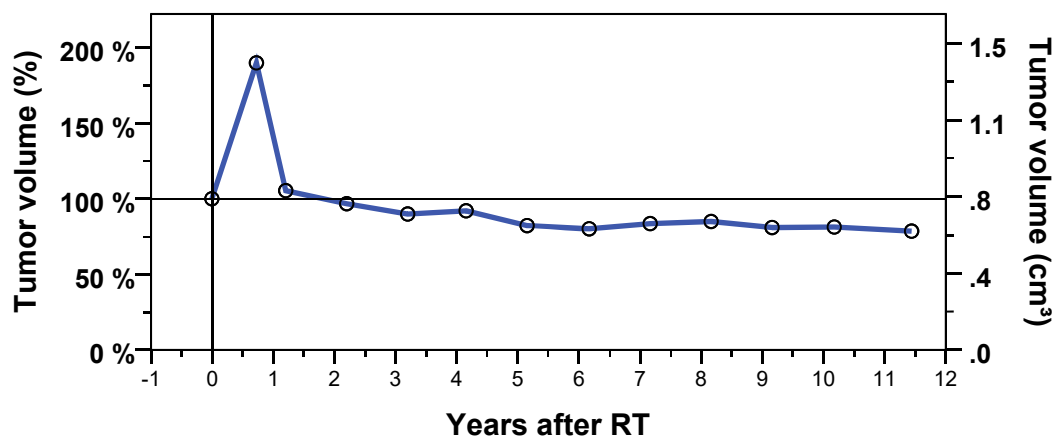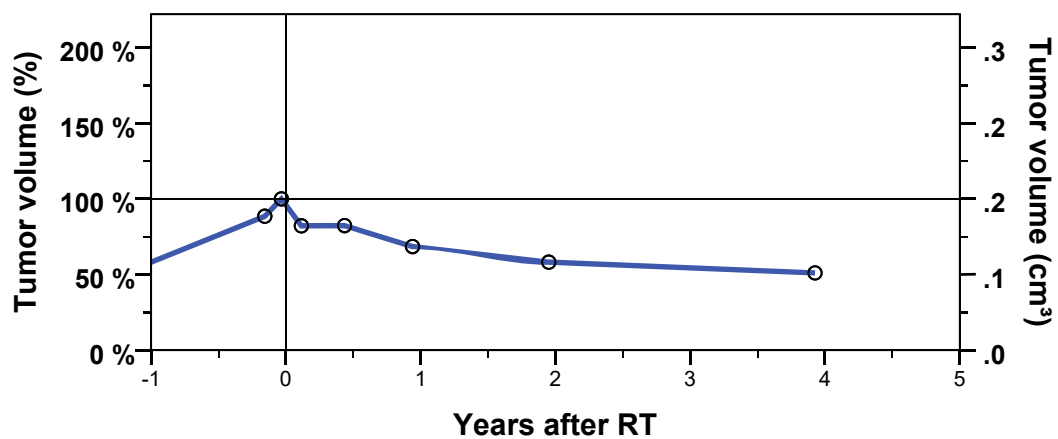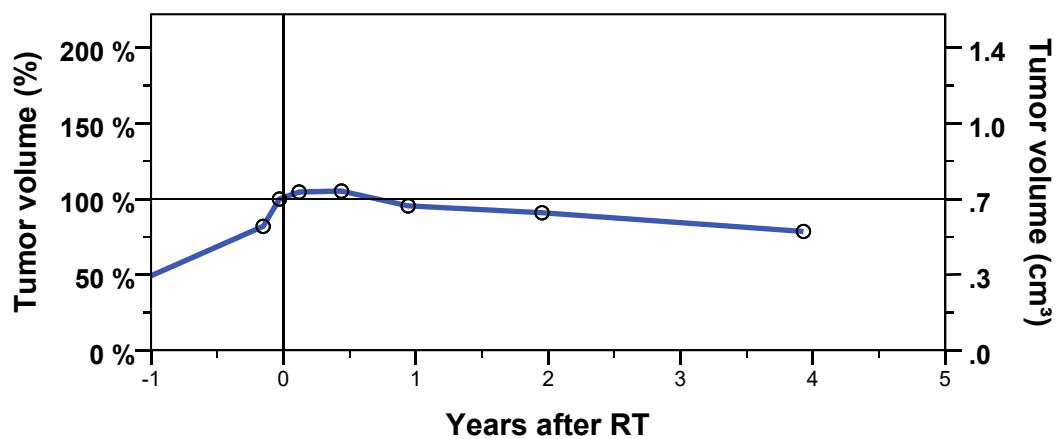

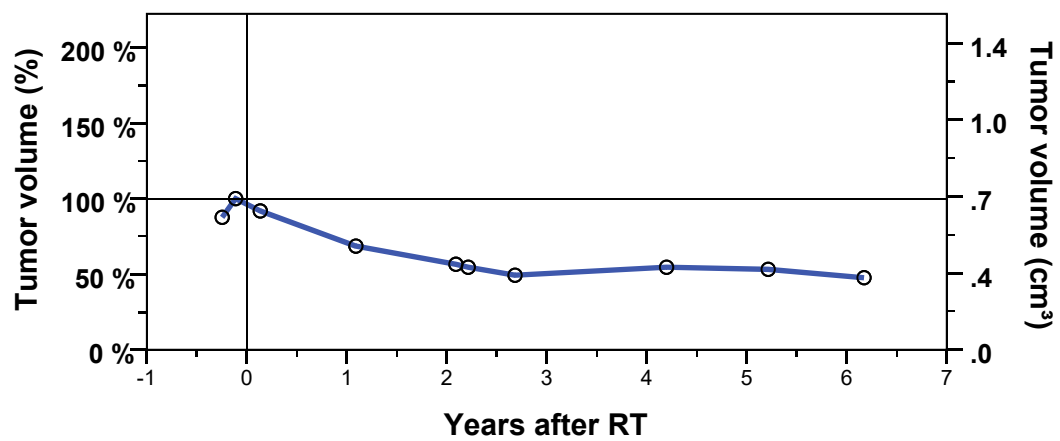

Supplement: Supplementary file 1 [file cancers-14-01547-s001.zip › cancers-1586877-supplementary.pdf]
